# Ensuring reliability of safety-critical clinical applications of computational cardiac models

**DOI:** 10.3389/fphys.2013.00358

**Published:** 2013-12-11

**Authors:** Pras Pathmanathan, Richard A. Gray

**Affiliations:** ^1^Center for Devices and Radiological Health, U.S. Food and Drug AdministrationSilver Spring, MD, USA; ^2^Department of Computer Science, University of OxfordOxford, UK

**Keywords:** modeling, software, verification, validation, uncertainty quantification

## Abstract

Computational models of cardiac electrophysiology have been used for over half a century to investigate physiological mechanisms and generate hypotheses for experimental testing, and are now starting to play a role in clinical applications. There is currently a great deal of interest in using models as diagnostic or therapeutic aids, for example using patient-specific whole-heart simulations to optimize cardiac resynchronization therapy, ablation therapy, and defibrillation. However, if models are to be used in safety-critical clinical decision making, the reliability of their predictions needs to be thoroughly investigated. In engineering and the physical sciences, the field of “verification, validation and uncertainty quantification” (VVUQ) [also known as “verification and validation” (V&V)] has been developed for rigorously evaluating the credibility of computational model predictions. In this article we first discuss why it is vital that cardiac models be developed and evaluated within a VVUQ framework, and then consider cardiac models in the context of each of the stages in VVUQ. We identify some of the major difficulties which may need to be overcome for cardiac models to be used in safely-critical clinical applications.

Mathematical and computational modeling is ubiquitous in the physical sciences and in engineering. One great challenge for the physiological sciences is to develop models of biological processes that are as credible, as predictive, and as useful as those used throughout physics and engineering. The great intricacy and variety of physiological models make this is a highly ambitious goal. However, reliable physiological models have the potential to provide a wealth of information for clinical decision making, treatment, and the development of medical products. In addition, we are entering a revolutionary new era of medicine, in which patient-specific genetic, anatomical and physiological information will facilitate early accurate diagnosis and patient-optimized therapy; translational research in the form of mechanistic computer models is expected to play a large role in this revolution. Accordingly, much effort has been expended in mechanistic physiological modeling, of which cardiac electrophysiological (CEP) modeling is one of the most advanced fields. It is now possible to run sophisticated whole-heart simulations using realistic anatomically-detailed geometries, predicting at high spatial and temporal resolution CEP activity from sub-cellular dynamics to the resultant electrocardiogram. Figure 1 illustrates the components that make up a CEP model. Electrophysiological models of the isolated cardiac myocyte are known as *cell models*, the first of which was proposed in the 1960s (Noble, 1962), building upon the pioneering Nobel Prize-winning work of Hodgkin and Huxley (1952). There are now over a hundred cardiac cell models, some predicting dozens of quantities such as: transmembrane potential; open channel probability of ion channel gates; currents corresponding to up to 25 channels, pumps and exchangers; and concentrations of various subcellular ionic species.

**Figure 1:**
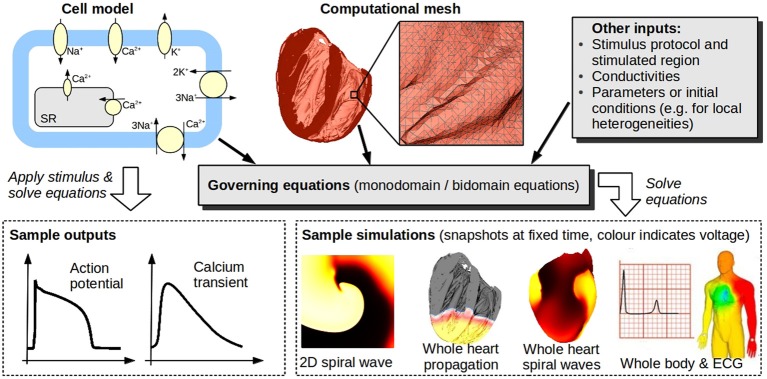
**Cardiac models**. Models of isolated cardiac cells (typically systems of ordinary differential equations) are known as cell models. Top left schematic illustrates channels, pumps, and exchangers modeled in a (relatively simple) cell model. Cell models can be used to predict quantities such as action potential, or they may be coupled to partial differential equations governing spatial propagation [usually the so-called monodomain or bidomain equations, see Keener and Sneyd (1998)], and, together with a computational mesh of the heart [(here, a high-resolution mesh of a rabbit heart (Bishop et al., 2010), comprised of 21 million elements, is shown)], used to simulate whole-heart EP activity. Heart models can be embedded in torso/body models to enable simulation of body surface potentials and ECGs. Bottom right images (body and ECG) taken from Zemzemi et al. (2013) reproduced with permission from *Wiley*.

Cardiac models have long been an integral tool for mechanistic investigation, generating and testing hypotheses, and designing experiments. That there are hundreds of publications involving modeling attest to the maturity and importance of modeling in the field. Currently, there is great interest in translating CEP modeling efforts to clinical settings, to aid in diagnosis and treatment. Potential applications attracting significant research interest include (Trayanova, 2012): (i) optimizing lead placement and waveforms for defibrillation therapy; (ii) identifying sites for ablation therapy to reduce cost and time in the clinical CEP lab and minimize damage to the heart; and (iii) optimizing lead placement for cardiac resynchronization therapy (CRT) (this application requires models of CEP coupled to cardiac mechanics and hydrodynamics). However, to use models as a clinical tool upon which safety-critical decisions are based, it is clear that the credibility and reliability of predictions will need to be thoroughly and rigorously evaluated. Whilst CEP modeling provides a powerful example of mechanistic insights gained via tight integration of modeling and experiment, the validity of these models in the clinical setting has yet to be established.

To determine the credibility of computational model predictions, the engineering and physical sciences communities have developed the field of *verification, validation and uncertainty quantification (VVUQ)*—often referred to as *verification and validation (V&V)*—which provides formalism, methodologies and best practices for evaluating the reliability of computational models (National Research Council, 2012). VVUQ has been a successful framework for enabling the use of models in numerous safety-critical fields. In this article we first argue that is it crucial that the cardiac electrophysiological community begins to develop and evaluate models within the context of VVUQ. We then discuss such evaluation of CEP modeling and each of the stages of VVUQ, and identify some of the major difficulties which may need to be overcome for models to be used in safely-critical clinical applications.

## A framework for assessing computational models

To present precise definitions of each term in VVUQ, we must distinguish between mathematical and computational models. A *mathematical model* is the underlying equations proposed to model a process, derived based on various simplifying assumptions. Mathematical models are usually too complex to solve analytically, so software is created to solve the mathematical model numerically, which is the *computational model*. Then:

- *Verification* is the process of ensuring that the computational model accurately solves the underlying mathematical model.
- *Validation* is the process of using data to evaluate the extent that the computational model accurately represents the real-world process which it attempts to simulate.
- *Uncertainty quantification* is the process of determining how uncertainty in inputs to the computational model (such as parameters and initial conditions) affect the results of the model.

**Figure 2:**
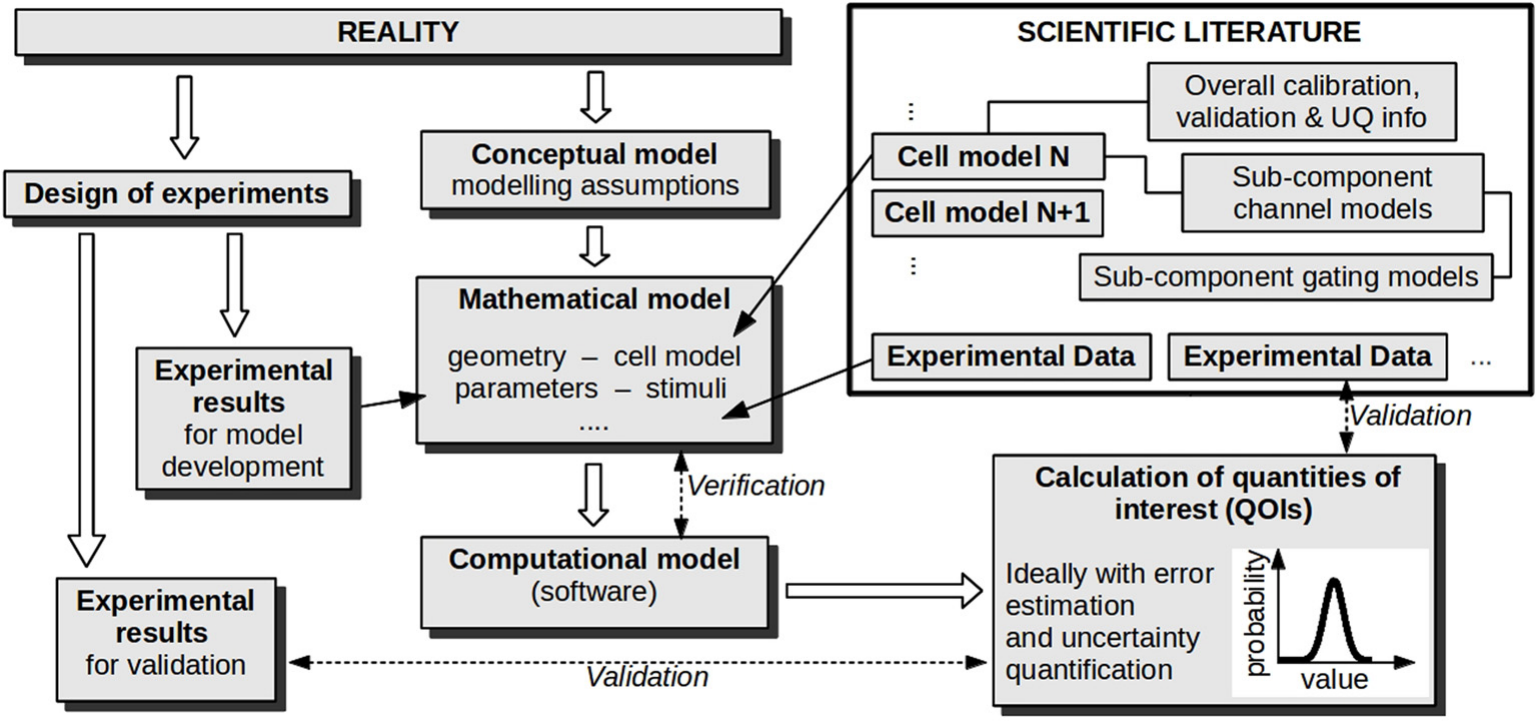
**Stages in developing and evaluating a model for a *particular cardiac application***. Verification and validation activities are labeled with dashed arrows. If uncertainty quantification is performed, parameter values are represented as probability distributions (not shown), and this uncertainty is propagated forward so that the calculated quantities of interest are also probability distributions. Note that the overall procedure can be iterative, where validation and UQ results are used to refine the model/experiments; this aspect is not illustrated.

The results of all of these stages are then used together to evaluate the credibility of model *predictions*, sometimes defined as simulations for which there is no corresponding data available. Figure 2 illustrates these stages for CEP applications.

VVUQ has been used extensively in engineering and the physical sciences. For example, the American Society of Mechanical Engineers (ASME) have produced a Guide for V&V in computational solid mechanics and a Standard for V&V in computational fluid dynamics and heat transfer (ASME, 2006, 2009, 2012). In recognition of the huge potential of twenty-first century computational modeling, the U.S. National Research Council was recently asked to compile a comprehensive report on VVUQ (National Research Council, 2012). One recommendation of this report, illustrating the belief that computational modeling will become a fundamental tool in twenty-first century science and the resultant importance of VVUQ, is that a basic understanding of VVUQ should be expected of the next generation of scientists as part of their core training. The use of modeling at NASA provides a further example of the importance of VVUQ, and also an example of the catastrophic consequences that are possible if the trustworthiness of models is not carefully established. The Columbia Disaster in 2003 is believed to be due, in part, to engineering decisions based on “incorrect” model predictions, as well as engineers not acting upon other model predictions where it was felt the trustworthiness was not clear (Columbia Accident Investigation Board, 2003; Sainani, 2012). As a result of the tragedy, NASA developed a comprehensive VVUQ-based standard for assessing their models (NASA, 2009). The Center for Devices and Radiological Health at the U.S. Food and Drug Administration (FDA) is currently developing a VVUQ-based framework for evaluating model results submitted as scientific evidence to support medical device regulatory submissions (Sainani, 2012). Reassuringly, VVUQ is also used by the nuclear modeling community (Harvego et al., 2010), amongst others. For detailed introductions to V&V/VVUQ, see for example Oberkampf et al. (2004), Oberkampf and Roy (2010), National Research Council (2012). For examples of VVUQ in practice, see for example ASME (2012), the case studies in National Research Council (2012), and Pathmanathan and Gray (2013).

VVUQ has, however, rarely been explicitly employed in cardiac modeling, partly due to a lack of knowledge on VVUQ, and because cardiac models are currently mostly used for hypothesis-generation, hence requiring less stringent evaluation of credibility. For models to be used in diagnostic or surgical guidance, incorrect predictions may have huge repercussions on patient-safety, public health and public confidence, as well as financial implications, and rigorous testing is clearly required before such guidance can take place. VVUQ provides a formal way to do this (Post and Votta, 2005), hence it is important that cardiac models begin to be evaluated in this context. Also, given the success of VVUQ in other fields, and the adoption of VVUQ in model-evaluation strategies by NASA and the FDA, a VVUQ-based assessment may be *expected* of proposed clinical cardiac models, at least in the U.S.

## Verification: does the software do what it is designed to do?

Let us consider cardiac modeling in the context of each stage of VVUQ, beginning with verification. The following is a brief discussion of verification of CEP models; for more details see Pathmanathan and Gray (2013). Verification involves confirming that a solver (software) correctly solves the equations it claims to solve. This is especially difficult with cardiac solvers involving propagation [i.e., tissue-level/whole-heart solvers, such as Vigmond et al. (2003), Bradley et al. (2011), Mirams et al. (2013)]. Verification involves attempts to minimize two types of error: (i) programming error, which can be avoided or identified using good software engineering practices, not discussed here; and (ii) numerical error, the difference between the computed solution and the true (generally unknown) solution of the equations. Numerical error is unavoidable, but should decrease to zero as the computational mesh resolution increases and time-steps decrease. Demonstrating that this is the case is one goal of verification, but is highly non-trivial. One approach for verifying solvers is to compare different solvers on a common, unambiguously-defined, “benchmark” problem. This is common in other fields [astronomy (Frenk et al., 2009), meteorology (Andren et al., 2006), seismic data processing (Hatton and Roberts, 1994)], but only recently has such a comparison been carried out for CEP solvers (Niederer et al., 2011). Surprisingly large differences between solvers were observed, later explained in Pathmanathan et al. (2012). To complement this test, Pathmanathan and Gray (2013) developed a range of complex but non-physiological cardiac EP problems for which the true solution is known, and can be used to help verify solvers. In general, however, more benchmark problems—both with and without known solutions—need to be developed, on which current and future tissue-level solvers can be rigorously tested.

The second part of verification is determining bounds on numerical error in the final *application*, i.e., the simulation used to make predictions and then base decisions. This should be performed for the specific output “quantities of interest” (QOIs) [such as action potential duration (APD), conduction velocity, or when re-entrant activity self-terminates] upon which the decision making will be based. Understanding the magnitude of numerical errors is very important, for the same reason that experimental measurements should be associated with uncertainties. However, determining such estimates may be extremely challenging in cardiac modeling, especially for simulations involving fibrillation (Pathmanathan and Gray, 2013).

## Validation: there is no such thing as a truly “validated model”

Validation involves the comparison of model simulations and experimental results. However, the goal should never be to declare a model “validated”; at best a model can be considered credible in precisely-defined “contexts of use” (National Research Council, 2012). Methods for comparisons of simulations and experiments range from simple “by-eye” qualitative comparisons, to more formal and quantitative approaches. More advanced approaches involve careful design of validation experiments (Oberkampf et al., 2004), together with statistical methods for comparing experiment and simulation, which fall broadly within two statistical philosophies: so-called “frequentist” and “Bayesian” approaches. Frequentist approaches to validation include Oberkampf and Barone (2006), which advocates careful development of “validation metrics”; Bayesian approaches include Kennedy and O'Hagan (2001), Wang et al. (2009), and involve accounting for *a priori* knowledge. The Bayesian approach can be much more complex statistically (e.g., Kennedy and O'Hagan, 2001), but can be extremely powerful, capable of uniting validation, calibration (defined later) and UQ (Kennedy and O'Hagan, 2001). As far as we are aware, no advanced methodology, either frequentist or Bayesian, has been utilized for CEP model validation.

Validation should involve comparison of the same QOIs in models and experiments. In physiology this is often hindered by difficulties in making direct experimental measurements of important quantities. Sometimes additional modeling is used to estimate a variable from indirect recordings of the model variable, introducing further complexity. In considering an improved paradigm for validation of cardiac models, it is also worth distinguishing between two distinct validation stages. As mentioned above, there are over a hundred cardiac cell models (see the CellML repository, www.cellml.org). An early stage in applying a cardiac simulation to a particular problem is choosing an appropriate cell model (see Figure 2). One may ask two validation questions: (V1) how much validation has been performed on that cell model (“how good is the chosen cell model in general?”); and (V2) how much validation was performed on the overall model for the proposed application (“how good is the overall tool, for this application?”). For example, if patient-specific whole-heart simulations were being used to optimize CRT lead placement, the ideal situation would be to choose a highly-validated and credible human cell model, embed it in whole-heart computations, and perform rigorous frequentist or Bayesian validation on the simulated QOIs (perhaps local activation times, or ejection fraction) that would be analysed and used by the physician in deciding lead placement. Certainly, the more rigorous the validation of the overall tool (V2), the better. In contrast, the level of validation required of the cell model (V1) is more debatable. In principle, performing validation of the overall tool might make validation of the cell model less necessary. However, to maximize the ability to an evaluator to make an informed decision on model credibility, it may be important that the strengths and weaknesses of the chosen cell model are made transparent, especially given the enormous complexity of these models. Currently, cell models show significant variability in predictions, and credibility is highly doubtful for many QOIs. The idea of *functional curation* (Cooper et al., 2011) may provide a solution to some of these limitations. Here, the aim is to develop the computational infrastructure that would allow a comprehensive set of virtual protocols to be automatically applied to existing, altered, or newly-developed cell models. This would allow immediate identification of quantities for which a cell model does and does not reproduce experiment results—a powerful tool for development, validation, and assessment of prediction credibility.

## Uncertainty quantification: where are the uncertainties and how are predictions affected?

All models contain parameters (which in this context includes initial conditions) which need to be measured or otherwise determined. However, parameter values are often associated with high levels of uncertainty. Many parameters will vary across a population or within an individual. Measurable parameters will suffer from inherent experimental uncertainty. Parameters which cannot be directly measured are often determined through *calibration* of a model, which typically provides a “best” or “most likely” value of a parameter, but again there is underlying uncertainty. Uncertainty quantification (UQ) involves characterizing uncertainty in the inputs to models, and determining how they affect the output QOIs. It is closely related to sensitivity analysis (SA), but whilst SA normally involves determining the effect of outputs to arbitrary variations in a chosen input, UQ makes direct use of pre-existing information on the uncertainty in inputs, usually in the form of probability distributions. The general aim of UQ is to use probability distributions to represent input parameters (rather than single values), and propagate these forward through the model to obtain probability distributions for resulting QOIs. This can be extremely computationally-demanding, but may be hugely more informative than performing one simulation with one parameter set and obtaining a single value of a QOI. Complete parameter uncertainty quantification may only be possible for simpler models with less parameters, but a simple model with UQ can be far more useful than a complex model with no UQ. UQ can help rigorously determine “most likely” parameter combinations, and also identify cases when multiple parameter combinations fit data equally well. For more details on UQ, see for example Kennedy and O'Hagan (2001), Roy and Oberkampf (2010).

Whilst SA has been performed in cardiac modeling (e.g., Sobie, 2009), the only example to our knowledge of true UQ in cardiac modeling is Elkins et al. (2013), where uncertainty in dose-response curves (of several compounds on four major ion channels) was characterized, and the resultant uncertainty in action potential computed. Comprehensive UQ is one of the major challenges in applying rigorous VVUQ to cardiac models. Complex cell models can involve hundreds of parameters, the majority obtained through calibration to experimental current-voltage relationships, usually with no information on uncertainty or variability. Systematically characterizing all the uncertainties, and propagating these forward to obtain uncertainties of QOIs, would demand huge effort and computational resources. A more tractable approach may be to use a mixture of scientific insight and sensitivity analyses to identify a set of important parameters for a given application, and perform rigorous UQ on these.

## Credibility of predictions and the need for transparency

After VVUQ has been performed, one aspect remains: using all available information to assess the *credibility of predictions in a specific application*. This assessment depends crucially on whether the prediction scenario is *interpolative* or *extrapolative* of the validation experiments. If the QOI being predicted is the same as that compared in the validation stage, and if the prediction scenario is in some sense contained within validation experiments (for example, suppose experimental validation of a simulated APD is performed at two pacing rates, and APD predicted at an intermediate rate), the prediction is interpolative. If not (e.g., prediction at a non-intermediate rate; or validation of action potential followed by prediction of calcium transients), it is extrapolative. Assessing extrapolative predictions is especially difficult. In this case there is no objective set of rules for evaluating credibility; instead a subjective assessment must be made based on (i) understanding of the model, (ii) physiological insight and (iii) VVUQ results. One issue currently prohibiting the first of these for CEP models is the unknown or variable origin of parameters within cell models—often entire sub-systems are taken or derived from previous cell models, and it can be difficult to trace back the origin of particular parameters. For example, Niederer et al. (2009) carefully identified “inheritance trees” for two major cell models, illustrating how model parameters originated from experiments covering a wide range of species and temperatures. In Figure 2, each cell model in the scientific literature is shown as directly linked to its supporting calibration and validation information, but in reality such information is not easily available. For the future, and specifically to facilitate informed assessment of extrapolative predictions, it is important that cell models become completely transparent, in particular that origins of parameters, calibration datasets, and validation results are all readily accessible, as well as models' assumptions, limitations and appropriate “contexts of use.”

## Final thoughts

Post and Votta (2005) recently argued that computational modeling is entering a very dangerous period. It is true that the consequences of inaccurate predictions in computational bio-medicine are extremely high. However, the potential long-term benefits of reliable models, including a revolutionary new era of patient-optimized clinical practice, are immense. To safely reach this goal, it is vital that knowledge established by the engineering and physical sciences, for developing and evaluating models used in safety-critical decision making, is taken advantage of. Applying such ideas and techniques to models of cardiac electrophysiology will be a huge challenge, but one with enormous potential rewards.

## Disclosure

The mention of commercial products, their sources, or their use in connection with material reported herein is not to be construed as either an actual or implied endorsement of such products by the Department of Health and Human Services.

### Conflict of interest statement

The authors declare that the research was conducted in the absence of any commercial or financial relationships that could be construed as a potential conflict of interest.
